# Polyphenolic Profile and Antioxidant Activity of Green Extracts from Grape Pomace Skins and Seeds of Italian Cultivars

**DOI:** 10.3390/foods12203880

**Published:** 2023-10-23

**Authors:** Massimo Guaita, Silvia Motta, Stefano Messina, Francesco Casini, Antonella Bosso

**Affiliations:** Consiglio per la Ricerca in Agricoltura e l’Analisi dell’Economia Agraria—Centro di Ricerca Viticoltura ed Enologia, Via P. Micca 35, 14100 Asti, Italy; silvia.motta@crea.gov.it (S.M.); stefano.messina@crea.gov.it (S.M.); casinifrancesco1996@gmail.com (F.C.); antonella.bosso@crea.gov.it (A.B.)

**Keywords:** grape pomace, green extraction, polyphenols, DPPH, fractionation

## Abstract

The possibility of industrial exploitation of winemaking products, as for all byproducts of vegetal origin, constantly deals with a raw material (grape pomace, GP) whose chemical composition and functional properties vary over time depending on the varietal and geographical origin of the grapes, the climatic conditions (vintage effect), and the winemaking technique. This work studied the compositional variability of polyphenolic skin and seed extracts from GP derived from white and red winemaking of different Italian grape varieties. The total polyphenolic content (GAE), the main classes of polyphenolic compounds, and the DPPH index were determined. Seed extracts were always richer in total polyphenols and condensed tannins and had higher antiradical activity (DPPH) than skin extracts: 144–298 mg GAE/g d.w. extract for skins and 327–540 mg GAE/g for seeds; the DPPH values were 1.77–3.40 mg AAE/g for skins and 3.10–10.48 mg AAE/g for seeds. Furthermore, it was verified that the evaluation of the GAE index of seed extracts, offering a good estimate of the antiradical properties (DPPH index), could represent a simple and rapid method for selecting the best lots of seeds to be used. Conversely, GP skins could be used as flour in the food industry due to their high content of dietary fiber and the presence of flavonols, which possess very interesting functional properties. Important differences in the flavonols profile were observed both between cultivars and between unfermented and fermented pomace.

## 1. Introduction

The world wine industry generates large quantities of byproducts annually, which are only partially reused. Quantitatively, the most important winemaking byproducts are grape pomace (GP) and stalks; in particular, the world annual production of GP has exceeded 10 Mt [1]. The disposal of GP can cause environmental problems such as pollution of ground and surface water, the attraction of disease-spreading vectors, and oxygen consumption in soil and groundwater, with consequent impacts on wildlife [2]. Large quantities of GP disposed in fields and vineyards after the harvest season can have negative effects on biodegradation due to the low pH and the presence of polyphenols that have antibacterial activity [3].

On the other hand, among agro-industrial byproducts, GP represents one of the richest sources of various compounds with beneficial effects on human health, in particular natural polyphenols and dietary fibers. Numerous alternative uses have been proposed for the valorization of GP, and the number of published works on this specific subject has been constantly increasing since 2013 [4].

From GP, it is possible to obtain GP flours (whole or as separated skins/seed fractions) that can be used for the production of functional food and dairy products [5], with the purpose of enriching foods with dietary fiber and total polyphenols, increasing antioxidant activity, protecting against lipid oxidation and, in some cases, improving the acceptability of the products [6].

It is possible to obtain polyphenolic extracts from GP flours, with higher added value and more potential applications than raw GP flours. Due to their antioxidant and antimicrobial action, these polyphenolic extracts can be used in the food industry to increase shelf life [7,8,9]. Dried polyphenolic extracts from GP are also used as oenological tannins (circular economy), particularly as processing adjuvants in the clarification of musts and wines, as additives for antioxidasic and antioxidant activity, stabilization of red wine color, and elimination of reductive off-flavors [10]. Other applications concern the cosmetic, pharmaceutical, and medical sectors [11].

More recently, natural polyphenols extracted from GP have been profitably used in material science for the surface functionalization of biomaterials (glass and titanium) for bone contact application due to their positive antimicrobial and tissue regeneration properties [12,13]. Polyphenols can also act as both reducing agents and stabilizers in the synthetic process for the achievement of metal-based oxide nanostructures [14,15].

Like all material of plant origin, GP has a variable composition linked to the vintage, the cultivar, the winemaking technique (white or red winemaking), the duration of maceration, and the subsequent drying and storage conditions. This variability of composition concerns both the overall quantity (purity) and the nature of the extractable phenolic compounds and affects its final application [16]. According to [17], despite a considerable number of studies on the possible applications of GP, they are often ineffective as they are not successfully implemented on a large scale. It is therefore necessary to encourage companies to implement processes that allow for the recovery and valorization of GP by deepening the knowledge of this precious byproduct. Considering the wide range of potential uses of polyphenolic extracts, the knowledge of how their polyphenolic composition varies according to the cultivar, the plant material of origin (skins or seeds), and winemaking techniques represent an aspect of particular interest in finding industrial uses [18]. This research was aimed at studying this variability in order to collect information on the potential, in terms of bioactive polyphenolic composition and antioxidant properties, of extracts obtained with a green process from GP derived from different cultivars and different winemaking processes (seeds and skins from fermented and unfermented GP).

## 2. Materials and Methods

Seven grape cultivars were studied (Table 1): 3 white cultivars (Muscat blanc, Arneis, Cortese) and 4 red cultivars (Barbera, Grignolino, Pinot noir, and Nebbiolo). GP was sampled from local wineries in the Piedmont area (Italy) during the 2020 harvest after pressing (fresh unfermented GP, from white winemaking process: UGP) or after racking off and pressing (fermented GP, from red winemaking process: FGP). As regards Pinot noir, both UGP and FGP were studied (from white and red winemaking, respectively), and Nebbiolo was sampled as FGP after macerations of different durations. The study focused on the composition of skins and seeds separately for each pomace sample.

### 2.1. Drying, Extraction, and Characterization of the Extracts

#### 2.1.1. Treatment and Drying of GP

The pomace of white cultivars and Pinot noir 1 is derived from white winemaking. It was sampled at the wineries after pressing the grapes before beginning alcoholic fermentation. Before drying, UGP samples were washed with water to remove residual sugars. The pomace of red cultivars was sampled at the end of fermentative maceration (racking off) after soft pressing (0.5 bar).

GP was subjected to pre-drying for 48 h at room temperature in a ventilated and dehumidified room, followed by drying at 40 °C in a ventilated oven for 48 h, up to constant weight (residual humidity 5–10%).

#### 2.1.2. Extraction

The seeds were manually separated from the skins, and the two fractions were milled in a coffee grinder. The extraction of polyphenols with ethanol:water (1:1) was performed according to our standardized protocol [19]; this green procedure allows for the complete recovery and reuse of the solvent. Moreover, ethanol itself is a byproduct of the wine industry. The use of ethanol instead of more extractive solvents was aimed at obtaining polyphenolic extracts intended for human consumption (enological tannins, food additives) or use in the medical/cosmetic/pharmaceutical field.

- 100 g of seeds or skins flour were extracted with a 1:10 *w/v* ratio in H_2_O/ethanol 50:50 *v/v* (1 L).
- Sonication for 20 min (50 W, 48 kHz ± 10%), then 2 h stirring with an orbital shaker.
- Centrifuge: 4000 rpm (2880× *g*), 18 °C, 20 min. A Centrifuge 5810 R Eppendorf (Hamburg, Germany) was used.
- 2 successive filtrations at 5 μm and 3 μm.

The filtered extracts were deprived of alcohol in rotovapor, then subjected to freeze-drying for 2 days at −50 °C (Labconco FreeZone^®^, LABCONCO Corporation, Kansas City, MO, USA).

#### 2.1.3. Polyphenolic Characterization of the Extracts

Approximately 20 mg of freeze-dried extract were redissolved in 3 mL of wine-like solution (tartaric buffer pH = 3.2, 13% *v*/*v* alcohol) for the determination of total flavonoids and total polyphenols (GAE) by spectrophotometry, with the same methods as reported by [19].

The total condensed tannins content, their mean degree of polymerization (mDP), and the percentage of each constitutive unit were determined with the phloroglucinolysis HPLC method [20]; the operating protocol of the method was the same as reported by [21].

Hydroxy cinnamyl tartaric acids (HCTA) were determined by HPLC according to [22]. The concentrations of each HCTA (both cis- and trans- isomers of caftaric, coutaric, and fertaric acids) were determined using six-point calibration curves obtained with pure standards (caffeic, coumaric, and ferulic acids, respectively). Each standard was injected in triplicate to assess both the linearity and repeatability of the method.

Flavonols were determined using the same chromatographic conditions and sample preparation as HCTA but with signal monitored at 360 nm [23]. The concentrations of each flavonol (quercetin, q. glucuronide, q. glucoside, kaempferol, k. glucuronide, k. glucoside) were determined using six-point calibration curves obtained with pure standards.

Gallic acid, monomer flavan-3-ols—(+)-catechin, (−)-epicatechin and (−)-epicatechin-3-O-gallate—, dimers B1, B2 and B3, and trimer C1 were determined by HPLC using a method for seed analysis [24] modified for wine. Samples were filtered with a 0.45 μm polypropylene filter (VWR International, Milano, Italy) and injected (20 μL). The separation occurs on an ODS Hypersil RP-C18 reversed-phase HPLC column (200 mm × 2.1 mm I.D., 5 μm packing, Thermo Scientific, Waltham, MA, USA) at 25 °C. The flow rate was 0.25 mL/min. Phase A was H_3_PO_4_ 10^−3^ M, and phase B was acetonitrile (HPLC grade). The signal was monitored at 280 nm, and the peaks were identified according to the external standard method. The concentrations of gallic acid and flavonols were determined using a six-point calibration curve obtained with pure standards of each molecule. Each standard was injected in triplicate.

#### 2.1.4. DPPH Test

The radical scavenging activity of the extracts was measured with the DPPH test: 30 μL of a solution of extract in methanol (approximately 25 mg of extract in 3 mL of methanol) was added to 2.97 mL of the DPPH reagent (stock solution: 6–7 mg DPPH in 25 mL methanol, sonicated for 10 min, filtered at 0.2 mm; then, the stock solution is diluted 10 times for the analysis). Absorbance was measured at 515 nm after a 4 h reaction at room temperature. The percentage inhibition (I%) was calculated according to the formula: I% = ((A_0_ − A)/A_0_) ×100, where A_0_ is the absorbance of the control without the sample, and A is the absorbance of the sample after a 4 h reaction. The I% values were transformed into ascorbic acid equivalents (AAE) expressed in g/L, according to the formula: AAE = I%/16,439, calculated using 5 standard solutions of ascorbic acid in methanol (concentrations of 0.05, 0.1, 0.2, 0.4, 0.5 g/L). The results were reported as mg of ascorbic acid equivalents per 1 g of freeze-dried extract (mg AAE/g d.w.).

### 2.2. Fractionation of the Freeze-Dried Extracts

The extracts were subjected to fractionation to verify the possibility of obtaining purified fractions of specific classes of polyphenolic compounds. The fractionation of the extracts was performed with C18 Sep-Pak cartridges, exploiting the different affinity of the molecules for the elution solvents used in succession, according to the method proposed by [25,26]:

- Two C18 Sep-Pak cartridges connected in series (top, Waters Sep-Pak Plus tC18 environmental cartridge; bottom, Waters Sep-Pak Plus tC18 cartridge) were washed with methanol (10 mL) and distilled water (20 mL), then they were activated with 10 mL phosphate buffer at pH 7.0 (67 mM);
- 25 mg of freeze-dried extract was dissolved in 20 mL phosphate buffer and loaded on the C18 cartridges;
- Elution with phosphate buffer (10 mL) → Fraction 1;
- Elution with ethyl acetate (25 mL) → Fraction 2;
- Elution with methanol (15 mL) → Fraction 3;
- Fraction 2 was vacuum-dried at 35 °C with a Genevac evaporator, redissolved in phosphate buffer (3 mL), and loaded again on the C18 cartridges. Fraction 2A was then eluted with ethyl ether (25 mL) and fraction 2B with methanol (15 mL).

All fractions were finally vacuum-dried at 35 °C with a Genevac evaporator and stored in a freezer at −20 °C.

A total of 4 fractions was obtained (1, 2A, 2B, and 3), in which the polyphenolic compounds were separated, indicatively, as follows:

Fraction 1: phenolic acids and HCTA, which were eluted with phosphate buffer at pH 7.0;

Fraction 2A: monomer flavan-3-ols, eluted in succession with ethyl acetate and diethyl ether;

Fraction 2B: oligomeric flavan-3-ols, eluted in succession with ethyl acetate and methanol;

Fraction 3: polymeric flavan-3-ols (proanthocyanidins or condensed tannins) eluted with methanol.

The total polyphenol content (GAE) was determined for all fractions using the same method reported in Section 2.1.3. Limited to fractions 2A and 2B, the monomer and oligomer flavan-3-ols content was determined, as reported in Section 2.1.3. Limited to fractions 2B and 3, the content and composition of condensed tannins were determined with the phloroglucinolysis HPLC method (Section 2.1.3).

### 2.3. Statistical Analysis

The data were processed with one-way analysis of variance (ANOVA) and Tukey’s test, and the correlation matrices between the different parameters describing the polyphenolic profile of the extracts and the DPPH_AAE_ parameter were calculated separately for skins and seeds. XLSTAT 2019 (Data Analysis and Statistical Solution for Microsoft Excel, Addinsoft, Paris, France, 2019) was used.

## 3. Results

### 3.1. Extraction Yields and Total Phenolic Content of Skin and Seed Flours

The extraction yields (g of freeze-dried extract obtained per 100 g of flour used) were variable among the different samples (Table 2) and ranged from 4.9% (Muscat blanc) to 15.6% (Pinot noir 1) for the skins and from 5.2% (Nebbiolo 2) to 17.5% (Pinot noir 1) for the seeds.

The extraction yields were, on average, lower than those reported by [27] for white GP of four different cultivars. In our work, a less performing “green” solvent (hydroalcoholic solution) was used, aiming to obtain and characterize polyphenolic extracts intended for human consumption or use in the medical/cosmetic/pharmaceutical field.

As regards skins, the highest extraction yield was observed for Pinot noir 1, followed by Nebbiolo 1 > Arneis, Barbera, Cortese, Grignolino, Pinot noir 2 > Nebbiolo 2, Muscat blanc.

Regarding seeds, the highest extraction yield was observed for Pinot noir 1, followed by Muscat blanc, Cortese > Nebbiolo 1, Arneis, Pinot noir 2 > Grignolino, Barbera > Nebbiolo 2. The extraction yield from seeds was more influenced by the winemaking technique: higher in seeds derived from non-macerated pomace (UGP) and lower in seeds from long-macerated GP (i.e., Nebbiolo 2).

The total polyphenolic content (GAE) of the skin and seed flours before the extraction was calculated from the GAE content of the freeze-dried extracts (reported in Table 3) according to the different extraction yields. The skin flours had GAE values ranging from 7.1 (Muscat blanc) to 26.2 (Pinot noir 1) mg/g d.w. of flour, while for the seed flours, the GAE values ranged from 12.6 (Nebbiolo 2) to 86.5 (Pinot noir 1) mg/g d.w. of flour. The polyphenolic content of the seeds was always higher than that of the respective skins.

The extraction yields from the seed flours were highly correlated with their total polyphenol content (r = 0.97), while for the skins, the correlation was lower (r = 0.81). Only about 65% of the variability in the extraction yields (R^2^ value of the linear regression equation) was explained by the polyphenolic content, while the remaining 35% depended on the content of other molecules. From some skin flours (Pinot noir 2, Grignolino, Arneis, and Cortese), with a lower polyphenolic content and proportionally lower extraction yields than Pinot noir 1 (the richest in polyphenols), the obtained extracts were richer in total polyphenols than the extract from Pinot noir 1, which probably had a higher content of extractable compounds other than polyphenols (Table 3). The lower correlation between GAE in the skin flours and the skin extracts could also be related to the presence of non-extractable polyphenols (NEPP) that remain in the residue after the extraction, permanently linked to dietary fiber. As we observed in a previous work [28], the NEPP content was higher in the skins than in the seeds. The concentration of NEPP in the skins varied with the cultivar, while the provenience as UGP or FGP did not seem to have a discriminating effect; conversely, the NEPP content of the seeds was more homogeneous among the different samples.

### 3.2. Polyphenolic Content of Freeze-Dried Extracts

The total polyphenols content (GAE) varied from 127 to 298 mg/g d.w. for the skins and 242 to 540 mg/g d.w. for the seeds (Table 3). The skin and seed extracts also differ in terms of total flavonoid content (108–322 mg/g d.w. in the skins and 303–754 mg/g d.w. in the seeds) and the condensed tannins content (59–143 mg/g d.w. in the skins and 158–414 mg/g d.w. in the seeds).

As regards the skin extracts, among white grape cultivars, Cortese and Arneis had a similar total polyphenols content (GAE), significantly higher than Muscat blanc; in addition, Cortese had a higher total flavonoids content than Arneis, linked to the higher condensed tannins content. Among red grape cultivars, the highest concentrations of total polyphenols, total flavonoids, and condensed tannins were observed for Pinot noir 2, followed by Grignolino.

As regards the seed extracts, significant differences in polyphenolic content were observed between white grape cultivars. Muscat blanc had the highest total polyphenols content (GAE), followed by Cortese and Arneis. On the other hand, Cortese extracts were the richest in condensed tannins and total flavonoids compared to the other two cultivars.

The polyphenolic content of Pinot noir seeds extract (as both UGP and FGP) fell within the concentration range of white grape cultivars. Considering the other red grape cultivars, Pinot noir was followed by Nebbiolo 1 > Grignolino > Barbera > Nebbiolo 2.

The richness in polyphenols of Pinot noir seeds is consistent with the data reported in the literature. The seed extracts from UGP (white cultivars and unfermented Pinot noir 1) were all significantly richer in polyphenolic compounds than those from FGP (excluding Pinot noir 2).

#### 3.2.1. Condensed Tannins Composition

The qualitative composition of condensed tannins in the extracts is reported in Table 3. The mDP varied from 4.8 (Pinot noir 1) to 7.9 (Cortese) in the skin extracts and from 3.4 (Pinot noir 1) to 6.6 (Cortese) in the seed extracts, with statistically significant differences between cultivars.

These mDP values are similar [29,30] or lower than those reported in the literature for skins and seeds from unfermented grapes ([31,32] for Pinot noir; [25,33] for red grape cultivars; [34] for white and red grape cultivars). The mDP values were also similar to or higher than those we observed in previous works [21,33] for skins and seeds from GP of the same red grape cultivars and similar to the values reported for GP by [27].

As regards the condensed tannins profile, (*−*)-epicatechin (EC) was the most abundant extension subunit both in the skins (from 46.0 to 65.0%) and in the seeds (from 52.1 to 63.0%). In most skin samples, the second most abundant extension subunit was (+)-catechin (C), followed by (*−*)-epicatechin-3-O-gallate (ECG), except for Arneis, Cortese, and Grignolino where ECG was more abundant than C. Conversely, ECG prevailed on C for all seed samples. Furthermore, (*−*)-epigallocatechin (EGC) was the lowest percentage subunit (from 0.8 to 5.5%) and was present only as an extension subunit in the skins. Among the terminal subunits, C prevailed over EC in the skin tannins and in most of the seed tannins (only in three cases did EC slightly exceed C). As regards the prevalence of EC both as an overall subunit and as an extension subunit and the prevalence of C as a terminal subunit, our data are consistent with those reported by [27] for condensed tannins extracted from GP.

The subunit profiles of the condensed tannins extracted from GP resulted differently from those reported for skins and seeds from whole fresh grapes. In the skins, EC (dominant subunit) is followed by EGC [25,31,34], while in the seeds, EC is followed by C or ECG, depending on the cultivar. An important percentage loss of EGC was observed in the fermented GP of some red cultivars compared to the corresponding fresh grapes [33], possibly due to its selective extraction during the fermentative maceration.

As regards the galloylation degree of condensed tannins (%ECG), the literature reports a marked difference between skins and seeds from whole fresh grapes. Seed tannins generally have a higher galloylation degree than skin tannins [31]. In the present work on GP, except for Muscat blanc, the differences in %ECG between seeds and skins were modest, and, in some cases, the skin tannins had, on average, higher %ECG than the seed tannins. These data are consistent with the results of our previous work focused on red grape cultivars [33]. For whole fresh grapes, the galloylated forms in seed tannins were 4.3 to 6 times higher than in skin tannins, but they dropped to 0.7–2.1 times for the respective fermented GP.

During the fermentative maceration and the subsequent pressing, a part of the tannins extracted from the seeds, rich in galloylated subunits, are probably selectively adsorbed on the skins. To our knowledge, this selective adsorption was never reported in the bibliography. In a previous work [35], we observed a higher adsorption of the trihydroxylated forms of anthocyanins compared to the dihydroxylated ones on the yeast’s cell walls during alcoholic fermentation. We already reported on the higher percentage losses during the fermentative maceration of the EGC subunits of skin tannins (trihydroxylated on the B ring) compared to C and EC (dihydroxylated). As regards the extracts from UGP, considering that it was not subjected to fermentative maceration but only to pressing, we could have expected the prevalence of the galloylated forms of tannins in the seeds compared to the skins, which, on the contrary, was observed only for Muscat blanc. However, it cannot be excluded that extraction/adsorption phenomena may already occur during crushing and pressing and during storage of GP before drying. In a previous work on the high-temperature drying of GP [19], we observed an increase in the polyphenolic content of the skins at the expense of the seeds.

#### 3.2.2. Monomeric and Oligomeric Flavan-3-ols

Table 4 reports the content of the flavan-3-ols identified and quantified in the extracts: (+)-catechin (C), (−)-epicatechin (EC), and (−)-epicatechin-3-O-gallate (ECG) among monomers, procyanidin B1, B2, and B3 among dimers, and trimer C1.

Monomers were the most abundant molecules, mostly in seed extracts (22–319 μmol/g d.w. for seeds, 4.8–24.8 μmol/g d.w. for skins). Significant differences were observed between the extracts of the different cultivars: Pinot noir’s skins and seeds (as both UGP and FGP) were the richest in monomeric flavan-3-ols. C was the most abundant molecule in most of the skin and seed samples. Its concentration always exceeded that of EC, except for Grignolino (skins and seeds) and Nebbiolo (skins). The predominance of C on EC was observed in some white GP (Macabeu and Parellada), while in others (Chardonnay and Premsal blanc), C and EC were at similar concentrations [27]. C prevailed over EC in both skins and seeds of Greek red and white grapes [36,37], and, apart from one case (Shiraz seeds), in all skins and seeds of red and white grapes studied by [38]. ECG was present in all seed extracts, while small amounts were quantified in six out of nine skin samples. In the literature, ECG was identified only in grape seeds [39], and its presence in GP skins could depend on adsorption phenomena occurring during winemaking or the subsequent stages of GP processing.

Like monomers, oligomeric flavanols were more abundant in seeds than in skins. As regards skins, dimer B1 was predominant in seven out of nine extracts, and dimer B2 in Pinot noir (as both UGP and FGP). Dimer B3 was overall the least important. It was identified and quantified in only four out of nine extracts. As regards seeds, a higher variability of composition was observed between the different cultivars, with a variable prevalence of one of the four analyzed oligomers.

The varietal variability of oligomeric flavanols is confirmed by the literature; in general, the prevalence of procyanidin B1 or procyanidin B2 is reported depending on the cultivar [27]. Only [38] observed the prevalence of dimer B3 in the skins of some red and white cultivars grown in warm climates.

#### 3.2.3. Flavonols

Table 5 reports the contents of the main flavonols identified and quantified in the skin extracts: quercetin, kaempferol, and their respective glucoside and glucuronide forms. Moreover, myricetin-3-O-glucoside was identified in the skins of Grignolino, Barbera, Pinot noir 1, and Nebbiolo 1, and free myricetin only in Nebbiolo 1 (data not reported). The presence of aglycon myricetin and its glucoside form only in some red GP agrees with [38], who identified 3-O-glycosides (glucuronide and glucoside) forms of myricetin only in the skins of red grapes. The authors of [40,41] identified in white grape skins only 3-O-glycosides based on kaempferol, quercetin, and isorhamnetin flavonoid structures.

Flavonols are absent in seeds: these compounds accumulate only in the grape skins with the role of protecting berries from light, particularly from UVB. Only the 3-O-glycosides forms of flavonols (3-O-glucosides, 3-O-galactosides, and 3-O-glucuronides) were identified in grapes. The presence of quercetin, kaempferol, and myricetin as aglycones in GP skins is due to the enzymatic hydrolysis of the 3-O-glycosides forms during the winemaking process [42]. Therefore, the aglycons quercetin and kaempferol were more abundant in the skins from FGP, subjected to macerations, while the 3-O-glycosides forms prevailed in the skins from UGP, separated from the must immediately after pressing.

Given the differences between UGP and FGP in the content of free and glycosylated forms of flavonols, the skins of the different cultivars were compared for the total flavonols content (sum of the single compounds), expressed in μmol/g d.w. (Table 5). Statistically significant differences were observed between all cultivars: the highest concentrations were observed for Arneis skins, followed by Muscat blanc > Barbera > Nebbiolo 1 > Pinot noir 2. The presence of flavonol makes the skins fraction of GP interesting (particularly in the food sector) due to their important bioactive properties (antiradical, anti-inflammatory, antioxidant, antiviral, antimicrobial, anticancer) [43].

#### 3.2.4. Phenolic Acids

Table 5 reports the content of gallic acid and hydroxycinnamyltartaric acid (HCTA) in the extracts.

Gallic acid was present in the skin and seed extracts at higher concentrations for seeds (except Grignolino and Nebbiolo 2). Muscat blanc seeds were those with the highest gallic acid content.

Modest concentrations of HCTAs, particularly trans-caftaric acid, normally the most abundant HCTA in grapes and wines [44], were detected, together with traces of other HCTAs. These acids are found mainly in grape juice but also in the skins, where the concentration ratios between some of the HCTAs have been used as varietal indices [45]. The total HCTA content in GP skins ranged from 0 (Pinot noir 1) to 5.3 µmol/g (Barbera); statistically significant differences linked to the cultivar and the kind of GP were observed. In addition, ref. [44] found significant differences between cultivars in the HCTA content of grape skins; since these differences varied with the vintage, the authors hypothesized the existence of a combined effect linked to the degree of ripeness of the grapes. Finally, t-caftaric acid was also identified in some seed samples (possible adsorption on the seed’s surface). Similarly, ref. [44] reported the presence of t-caftaric and t-cutaric acid in GP seeds.

### 3.3. DPPH Index

The antiradical capacity was significantly higher for seeds than skins (Table 3). However, this difference did not depend only on the higher polyphenolic content of the seeds, as can be seen from the comparison between the average values of the angular coefficients of the lines passing through the origin, which model the relationship between DPPH_AAE_ and GAE, respectively, equal to 0.0129 for the skins and 0.0172 for the seeds. At equal GAE values, the seeds had a higher antiradical capacity (DPPH) than the skins. This result could depend on the fact that condensed tannins, proportionally more abundant in seeds than in skins, have a higher antiradical activity than other classes of polyphenols [46].

#### Correlations between the DPPH Index and the Main Polyphenolic Compounds

Table 6 shows the correlation matrices between the DPPH_AAE_ index and the main parameters describing the polyphenolic profile of the extracts (as classes of polyphenolic compounds: total polyphenols, total flavonoids, condensed tannins, total monomeric and total dimeric flavan-3ols, total HCTA and total flavonols; as single compounds: gallic acid and trimer C1).

The correlation between DPPH and the main polyphenolic indices (total polyphenols, condensed tannins, and total flavonoids) was overall higher for seeds than for skins. This probably depends on the fact that seed polyphenols are mainly composed of flavan-3-ols (monomers and polymers), while skin polyphenols also include HCTA and flavonols, and this confirms the existence of a structure–activity relationship of the polyphenolic constituents in the different samples [47].

Overall, the highest correlations concerned the DPPH parameter and the GAE index. As regards polyphenolic compounds, a high correlation was observed between monomeric flavan-3-ols and dimeric flavan-3-ols in both the skin and seed extracts, while condensed tannins were correlated to monomeric and dimeric flavan-3-ols only in seed extracts. Finally, in the skin extracts, the HCTAs were correlated with gallic acid, while total flavanols were not correlated with any of the other parameters.

The relationship (univariate linear regression) between the DPPH parameter and each of the main polyphenolic compounds was also studied. Table 7 shows the regression lines between DPPH (variable Y) and each class of compound considered (variable X) separately for skin and seed extracts. The regression lines between DPPH and GAE were those with the best modeling capacity (highest *R*^2^).

**Figure 1:**
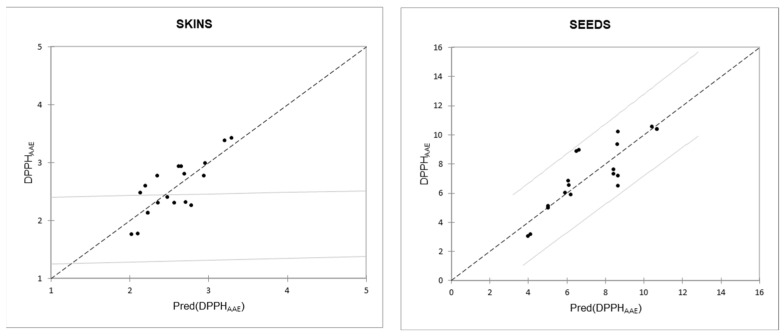
Relationship between measured and predicted values of the DPPH_AAE_ parameter, respectively, from the regression lines DPPH_AAE_ = 0.048 × Flavonols + 0.03 × Condensed Tannins + 1.106 (for skins) and DPPH_AAE_ = 0.962 × gallic acid + 0.015 × Condensed Tannins + 1.106 (for seeds).

Considering all classes of polyphenolic compounds, the multiple linear regression lines that best model the relationship with the DPPH parameter (variable Y) are those described by the variables “condensed tannins” and “total flavonols” for the skin extracts and those described by the variables “condensed tannins” and “gallic acid” for the seed extracts (Figure 1); the relationship between DPPH and GAE has R^2^ values close to those of the two aforementioned regression lines (Table 7).

### 3.4. Fractionation of Freeze-Dried Extracts

The fractionation was aimed at separating oligomeric and polymeric flavan-3-ols (proanthocyanidins) according to their molecular weight: monomers (Fraction 2A), oligomers (Fraction 2B), and polymers (Fraction 3). Fraction 1 was not considered since it did not contain flavonoids.

**Figure 2:**
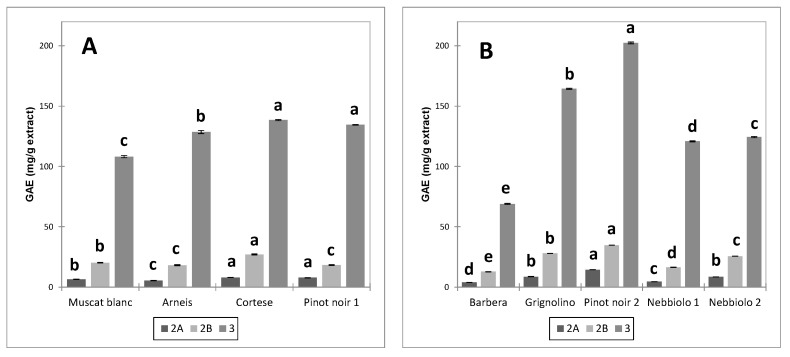
Average total polyphenols content (GAE) ± standard error in the fractions of the skin extracts ((**A**) = UGP; (**B**) = FGP) and ANOVA results. Different letters discriminate the samples significantly different from one another within each fraction (*p* < 0.05, Tukey’s test).

**Figure 3:**
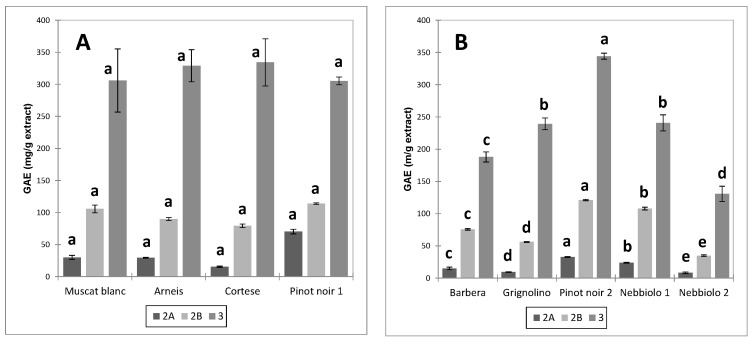
Average total polyphenols content (GAE) ± standard error in the fractions of the seed extracts ((**A**) = UGP; (**B**) = FGP) and ANOVA results. Different letters discriminate the samples significantly different from one another within each fraction (*p* < 0.05, Tukey’s test).

The distribution of total polyphenols (GAE) in the different fractions is reported in Figure 2 (skin extracts) and Figure 3 (seed extracts).

Fraction 3 was the richest in GAE, followed by fraction 2B and fraction 2A. The order was unchanged for all extracts. These data are consistent with the findings of [25] for wine and grape skins and seeds. The total polyphenols content of the seed extracts exceeded that of the skin extracts for all fractions. Significant differences were observed due to the cultivar and the winemaking technique (UGP or FGP).

As regards unfermented pomace (UGP), the extracts from Cortese and Pinot noir skins had the highest content of total polyphenols (GAE) in fraction 3 compared to Arneis and Muscat blanc. In proportion to the other cultivars, the total polyphenol content in fraction 2 of Pinot noir (PN1) was lower. Among the fermented pomace (FGP), the highest content of polyphenolic compounds in the three different fractions was observed for Pinot noir (PN2), followed by Grignolino, Nebbiolo (Ne2 and Ne1), and finally by Barbera, with statistically significant differences between all cultivars for fractions 2 and 3. In this case, no variations were observed in the proportions of the polyphenolic content between fractions, with the exception of fraction 2A, the least abundant.

Conversely, no statistically significant differences were detected between the fractions of seed extracts from UGP. As regards FGP seeds, the polyphenolic content of fraction 3 dropped significantly in the order Pn2 > Ne1 = Gr > Ba > Ne2.

In the case of skins, it is interesting to notice how the polyphenolic profile of the three fractions was superimposable for the two Nebbiolo samples derived from macerations of different duration (Figure 2b), while this did not happen for the respective seeds (Figure 3b), where the polyphenolic concentration of the three fractions was lower for Nebbiolo 2 (subjected to long maceration) than for Nebbiolo 1 (short maceration). This evidence confirms that with the prolongation of fermentative maceration, the extraction mainly concerns the polyphenolic component of the seeds (delayed extraction due to the presence of ethanol), while in the case of the skins, the losses in polyphenolic compounds due to extraction are counteracted by adsorption phenomena.

#### 3.4.1. Oligomeric and Polymeric Flavan-3-ols in the Skin Extracts

Table 8 reports the content of total polyphenols and condensed tannins in fractions 2B and 3 of the skin extracts.

The highest recoveries of condensed tannins were observed in fraction 3 (between 31.3 and 67.0% of the condensed tannins content in the total extracts), while in fraction 2B, the recoveries were more modest (4.6–11.0%). The concentrations of total polyphenols (GAE) and condensed tannins in fractions 3 and 2B were proportional to the respective concentrations in the total extracts (values of the correlation coefficients between the total extract and fractions 3 and 2B, respectively, equal to 0.934 and 0.903 for the GAE index and 0.937 and 0.921 for condensed tannins).

The fractionation separated the condensed tannins according to the different sizes. Fraction 2B contained oligomeric proanthocyanins with mDP values ranging from 2.2 to 3.0, while fraction 3 contained polymeric proanthocyanins with mDP values ranging from 7.3 to 10.4 (1.6 to 3.3 units higher than those observed in the total extracts).

The percentage weights of the different subunits (total, terminal, and extension subunits) that make up the polymeric proanthocyanins of fraction 3 were very similar to those observed for the total extracts (highly significant correlations and r values between 0.709 and 0.950). The same was not observed for the percentage weights of oligomeric proanthocyanins (fraction 2B), for which the correlation coefficients were lower and variable among the different subunits (r values ranging from −0.196 to 0.925).

The main differences in the subunit profiles between polymeric (fraction 3) and oligomeric (fraction 2B) proanthocyanins concerned the percentage weight of the terminal units, which was higher in fraction 2B (tannins with lower mDP). Overall, apart from some exceptions, the percentage weight of the C subunit was, on average, lower for polymeric proanthocyanins (fraction 3) than for oligomeric ones (fraction 2B); conversely, the percentage weight of the EC and G subunits was, on average, higher in fraction 3 than in fraction 2B.

#### 3.4.2. Oligomeric and Polymeric Flavan-3-ols in the Seed Extracts

Table 9 reports the content of total polyphenols and condensed tannins in fractions 2B and 3 of the seed extracts.

In addition, in the case of seed extracts, polymeric proanthocyanidins (fraction 3) were more abundant (from 35 to 52% of the condensed tannins content in the total extracts) than oligomeric ones (fraction 2B, from 10.6 to 24.1% of the condensed tannins content in the total extracts). The mDP of oligomeric proanthocyanins (2B) varied between 2.9 and 3.5 units, and these values were similar and homogeneous among the different cultivars, as already observed for the skin extracts. The polymeric proanthocyanins (fraction 3) had mDP values ranging from 6.7 to 10.4 units (2.8 to 4.8 units higher than those of the total extracts).

Compared to the total extract, the subunit composition of oligomeric and polymeric proanthocyanins in the two fractions changed. In fraction 2B, the percentage weight of C increased, while the opposite was observed for fraction 3. As already observed in other works [21,48], the condensed tannins with a lower mDP were richer in C than those with a higher mDP. In both fractions (2B and 3), the percentage weight of the galloylated forms decreased compared to the total extract.

In fraction 2A of the skin extracts, the B2 dimer was detected in trace amounts, while in fraction 2A of the seed extracts, the B1, B2, and B3 dimers and the C1 trimer were detected in quantifiable concentrations, only for some cultivars (data not reported).

As regards monomer flavan-3-ols, they were identified in fraction 2A in the skins and, only for some cultivars, were detected in trace amounts in fraction 2B. In the seeds, where their concentration is higher, they were predominantly present in fraction 2A and at very low concentrations (0–1 mg/g dw as catechin) also in fraction 2B. The monomer flavan-3-ols content in fractions 2A is reported in Table 10. For both skins and seeds, the content of (+)-catechin and (−)-epicatechin in fraction 2A was significantly correlated to the content of the same molecules in the respective extracts. The correlation coefficients (Pearson’s r) are, respectively, equal to 0.95 and 0.89 for C and EC in the skins and 0.98 and 0.91 for C and EC in the seeds.

### 3.5. PCA Analysis

**Figure 4:**
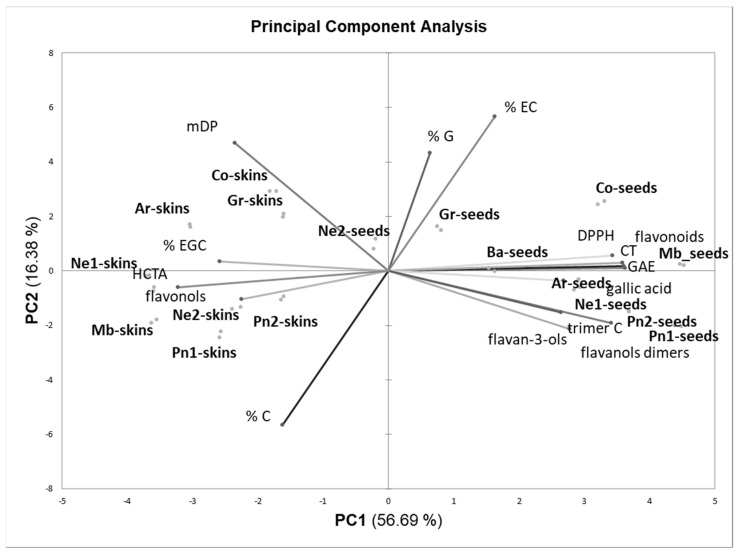
Loadings and scores for skin and seed extracts in the space defined by the first 2 Principal Components. Legend: Mb—Muscat blanc, Ar—Arneis, Co—Cortese, Ba—Barbera, Gr—Grignolino, Pn1—Pinot noir 1, Pn2—Pinot noir2, Ne1—Nebbiolo 1, Ne2—Nebbiolo2.

The data related to the polyphenolic composition of skin and seed extracts were finally subjected to PCA. Figure 4 shows the loadings (variables) and scores (samples) in the space defined by the first two Principal Components, which together describe 73% of the total data variability.

The first Component discriminates the seed extracts from the skin extracts. Compared to skins, seeds were characterized by higher polyphenolic content (GAE, total flavonoids, condensed tannins, flavonols, gallic acid, oligomeric compounds), lower HCTA content, and the absence of flavonols. The highest variability was observed for the seed extracts. The seeds from UGP (Mb, Co, Ar, and Pn1) were, on average, richer in polyphenolic compounds than those from FGP, with the exception of Pinot noir 2. A higher uniformity was observed for the skin extracts. The second Component discriminates the extracts according to the composition of condensed tannins: in particular, the skin and seed tannins of Grignolino and Cortese were richer in galloylated units.

**Figure 5:**
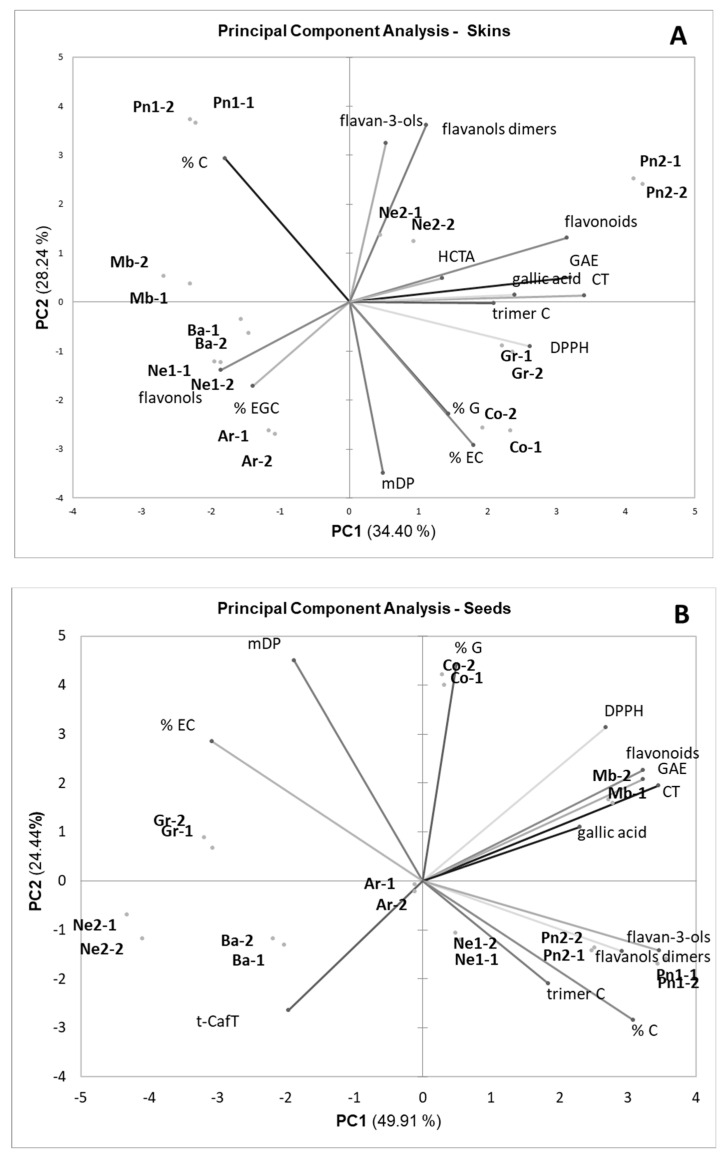
Loadings and scores for skin (**A**) and seed (**B**) extracts in the space defined by the first 2 Principal Components. Legend: Mb—Muscat blanc, Ar—Arneis, Co—Cortese, Ba—Barbera, Gr—Grignolino, Pn1—Pinot noir 1, Pn2—Pinot noir2, Ne1—Nebbiolo 1, Ne2—Nebbiolo2.

The PCA was repeated separately for skin and seed extracts (Figure 5). Figure 5A shows the distribution of the skin samples. The skins of Pinot noir 2 (FGP), Grignolino (FGP), and Cortese (UGP) were the richest in total polyphenolic compounds (GAE, total flavonoids, condensed tannins) and had the highest anti-radical activity (DPPH).

The origin of GP (FGP or UGP, white or red cultivars) was not a discriminating factor for skin extracts as observed for seed extracts. Unlike what was expected and what was observed for skin flours, the extracts from unfermented Pinot noir skins (PN1, UGP) were poorer in polyphenolic compounds than those from fermented skins (PN2, FGP) (differences in extraction yields, paragraph 3.1). Furthermore, Nebbiolo 2 skins, macerated for a longer duration, were richer in polyphenols than Nebbiolo 1 skins, macerated for shorter times (adsorption on the skins of the polyphenols solubilized in the must—wine).

It is also interesting to notice how the antiradical activity (DPPH parameter) was positively associated with the total content of polyphenolic compounds (total flavonoids, GAE, condensed tannins) while it was independent or negatively correlated with the content of single classes of polyphenolic compounds, respectively, monomeric and dimeric flavanols, and flavonols. This result confirms what was reported by [47], who observed the presence of correlations between the total polyphenols content (GAE) and the anti-radical activity determined with the DPPH test for GP seed extracts, particularly rich in condensed tannins, unlike onion skin extracts, richer in flavonols, which were more reactive towards the hydroxyl radical. Other authors observed that the antioxidant activity of the extracts was positively correlated with the mDP of condensed tannins up to mDP 10, while it dropped and remained at a lower level with mDP > 10 [49]. In our case, we observed that for the seeds, in which the percentage weight of condensed tannins in the total polyphenolic content was higher than for the skins, each concentration unit of total polyphenols (GAE) corresponds to a higher value of antiradical activity (DPPH).

As regards seeds (Figure 5B), the main differences in concentration (first Principal Component) were linked to the cultivar (Pinot noir was the richest) and also to the winemaking technique: UGPs were richer than FGPs. In the case of Nebbiolo, the polyphenolic content dropped in the seeds subjected to longer macerations (lower in Nebbiolo 2 than in Nebbiolo 1). As observed for the skins, the DPPH parameter was positively associated with the overall polyphenolic content (GAE, total flavonoids, condensed tannins) but not with the content of the individual classes of flavonols (monomers, dimers, and trimers).

## 4. Conclusions

The exploitation of the antioxidant and antiradical properties of polyphenolic extracts from GP depends on the quality of the raw material used. Many works in the literature have addressed this topic.

The obtained results confirm the outcomes of our previous experiences and other works in the literature and provide useful information for the exploitation of these byproducts.

In particular, we observed the following:

- More concentrated polyphenolic extracts can be obtained from GP seeds than from GP skins for different reasons:
  - when using skins from unfermented white GP (white UGP), the original whole grape skins were already typically poorer in total polyphenols than the respective seeds;
  - with skins from unfermented red GP (red UGP, i.e., Pinot noir), the extraction yields of the polyphenolic fraction from the flours with hydroalcoholic mixtures are lower due to the presence of a significant fraction of polyphenols bound to the fiber, which it is not extracted by these solvents;
  - skins from fermented red GP (red FGP) have lost a significant amount of phenolic compounds during the maceration process (red winemaking). In this last case, a longer maceration does not necessarily imply a lower content of polyphenolic compounds in the skins and in the relative extracts because, with the prolongation of the maceration, the adsorption on the skins of the polyphenolic compounds of the wine compensate for the losses due to extraction. On the contrary, for the seeds, the adsorption of polyphenols during maceration is lower (comparison between Nebbiolo 1 and Nebbiolo 2).
- The condensed tannins of the extracts from GP skins and seeds are much more similar to each other both in terms of mDP and monomeric composition compared to what is observed for grape tannins. As already observed above, this evidence depends on the fact that during maceration (red winemaking), a part of the skin’s polyphenols is lost by extraction and, at the same time, a part of the seed’s polyphenols is adsorbed on the skins. The opposite phenomenon (adsorption of skin polyphenols on the seeds) is modest due to the smaller adsorbent surface of the seeds.
- The antiradical capacity of the extracts (DPPH) is positively correlated to the total polyphenol content (GAE). Seed extracts, however, have a higher antiradical activity than skin extracts at the same concentration of total polyphenols (GAE). It is probably the content of condensed tannins, prevalent in the seeds compared to the other classes of polyphenolic compounds, that influences the antioxidant and antiradical properties of the extracts.
- When the aim is to obtain extracts with high antiradical capacity (for example, in medicine/pharmacology), the use of seeds should, therefore, be favored. Among the seed extracts, we observed that those richest in polyphenols (GAE) and with the highest antiradical properties were derived from Muscat blanc and Cortese (UGP) and Pinot noir (both UGP and FGP).
- On the other hand, GP skins could be advantageously used as flour in the food sector due to their high dietary fiber content. In this regard, the most interesting cultivars for polyphenolic content of the skins were Grignolino and Pinot noir.
- A high variability was observed in the polyphenolic content and the antioxidant and antiradical properties of the extracts analyzed. The GAE index is significantly correlated with the DPPH index. Therefore, it can be used as a rapid method for the characterization, selection, and blending of seed extracts of different origins in order to obtain extract batches that are homogeneous over time and have composition and properties suitable for different uses.
- Skin extracts are particularly rich in flavonols, molecules absent in seeds. To date, this class of compounds has been little studied in winemaking byproducts. However, these are molecules of particular interest, with important bioactive properties (antiradical, anti-inflammatory, antioxidant, antiviral, antimicrobial, anticancer), which can give added value to GP skin flours and extracts.

## Figures and Tables

**Table 1:** Description of the different grape pomace samples collected in local wineries.

	Cultivar	Type of GP	Characteristics
white cultivars	Muscat blanc	UGP	unfermented
	Arneis	UGP	
	Cortese	UGP	
red cultivars	Barbera	FGP	fermented
	Grignolino	FGP	fermented
	Pinot noir 1	UGP	unfermented
	Pinot noir 2	FGP	fermented
	Nebbiolo 1	FGP	fermented, short maceration
	Nebbiolo 2	FGP	fermented, long maceration

**Table 2:** Extraction yields and total polyphenols content (GAE) of the skin and seed flours. ANOVA results.

	Skins		Seeds	
	Extraction Yield (%)	GAE	Extraction Yield (%)	GAE
Muscat blanc	4.9 e ^a^	7.1 h	14.3 b	77.4 b
Arneis	7.4 c	15.5 c	11.3 c	51.2 e
Cortese	6.8 c	14.2 d	14.0 b	68.8 c
Barbera	7.0 c	8.9 g	9.2 d	30.0 h
Grignolino	6.6 cd	15.5 c	10.3 cd	35.8 g
Pinot noir 1	15.6 a	26.2 a	17.5 a	86.5 a
Pinot noir 2	6.5 cd	19.4 b	11.3 c	55.8 d
Nebbiolo 1	8.8 b	13.6 d	11.4 c	42.0 f
Nebbiolo 2	5.3 de	10.6 e	5.2 e	12.6 i
F	160	39,869	125	63,640
Sign	*** ^b^	***	***	***

The GAE index is expressed as mg/g d.w. flour. ^a^ Different letters along the column discriminate the samples significantly different from one another (*p* < 0.05, Tukey’s test). ^b^ Significance: *** represent significance at *p* ≤ 0.001.

**Table 3:** Total polyphenols (GAE) and total flavonoids content, polyphenolic profile of condensed tannins, and DPPH_AAE_ parameter of the freeze-dried extracts. ANOVA results.

					Condensed Tannins: Phloroglucynolysis												
							Total Monomeric Composition				Extension Units				Terminal Units		
		GAE	Total Flavonoids	DPPH_AAE_	Condensed Tannins	mDP	% EGC	% EC	% C	% ECG	% EGC-p	% EC-p	% C-p	% ECG-p	% EC	% C	% ECG
**Skins**	Muscat blanc	144 g ^a^	108 f	2.36 bc	64 ef	5.7 d	1.4 de	66.2 e	33.8 a	12.3 e	1.4 de	49.9 d	21.6 a	9.6 g	2.7 e	12.2 b	2.7 bcd
	Arneis	208 c	114 f	2.93 ab	72 de	6.6 c	5.1 b	85.9 a	14.1 e	12.3 e	5.1 b	64.8 a	4.9 f	10.1 fg	3.7 d	9.2 d	2.3 f
	Cortese	208 c	206 c	2.53 bc	108 b	7.9 a	1.0 f	85.5 a	14.5 e	22.2 a	1.0 f	60.1 b	7.7 d	18.5 a	2.2 e	6.8 f	3.7 a
	Barbera	127 h	190 d	2.40 bc	59 f	5.8 d	1.3 e	70.4 d	29.6 b	17.8 b	1.3 e	46.0 f	20.6 a	14.9 b	5.4 b	8.9 d	2.9 bc
	Grignolino	236 b	256 b	2.88 ab	134 a	6.8 bc	1.5 d	86.4 a	13.6 e	15.2 c	1.5 d	65.0 a	6.3 e	12.6 c	4.7 c	7.3 e	2.6 cde
	Pinot noir 1	169 e	175 d	1.77 c	64 ef	4.8 f	1.3 de	65.5 e	34.5 a	10.3 f	1.3 de	48.4 e	21.1 a	8.1 h	5.4 b	13.4 a	2.3 f
	Pinot noir 2	298 a	322 a	3.40 a	143 a	5.2 e	0.8 f	73.3 b	26.7 d	13.5 d	0.8 f	53.3 c	15.8 c	11.0 de	5.6 b	10.9 c	2.6 def
	Nebbiolo 1	154 f	137 e	2.35 bc	81 d	7.2 b	5.5 a	71.8 c	28.2 c	13.6 d	5.5 a	50.0 d	19.2 b	11.3 d	2.8 e	8.9 d	2.3 ef
	Nebbiolo 2	201 d	190 d	2.53 bc	97 c	4.9 ef	2.8 c	70.5 cd	29.5 bc	13.3 d	2.8 c	47.4 e	18.8 b	10.4 ef	7.0 a	10.7 c	3.0 b
	F	8978	642	9.0	351	239	2588	1244	1244	498	2588	1414	1078	582	215	654	58
	Sign	*** ^b^	***	**	***	***	***	***	***	***	***	***	***	***	***	***	***
**Seeds**	Muscat blanc	540 a	712 b	10.48 a	340 c	4.4 c	nd	79.6 c	20.4 b	20.1 b	nd	52.1 e	9.2 a	16.2 b	7.5 d	11.2 cd	3.9 ab
	Arneis	455 d	664 c	8.91 b	273 d	3.9 cde	nd	83.6 b	16.4 c	9.2 f	nd	62.3 a	5.7 e	6.6 f	12.1 a	10.7 de	2.6 e
	Cortese	492 c	754 a	9.77 ab	414 a	6.6 a	nd	85.4 a	14.6 d	22.8 a	nd	57.8 b	8.4 ab	18.6 a	4.8 e	6.1 g	4.2 a
	Barbera	327 g	492 e	5.95 de	221 e	4.0 cd	nd	83.7 b	16.3 c	12.7 e	nd	59.2 b	6.0 e	9.7 e	11.8 a	10.4 e	2.9 de
	Grignolino	348 f	501 e	5.06 e	256 d	5.7 b	nd	85.7 a	14.3 d	15.3 c	nd	63.0 a	7.4 cd	12.0 c	7.5 d	6.9 g	3.2 cd
	Pinot noir 1	497 b	680 c	6.85 cd	401 a	3.35 e	nd	77.0 d	23.0 a	13.5 d	nd	52.4 de	5.4 e	10.0 e	11.1 ab	17.6 a	3.5 bc
	Pinot noir 2	494 bc	714 b	7.47 c	374 b	3.50 de	nd	77.3 d	22.7 a	12.9 de	nd	54.3 cd	7.4 cd	9.7 e	10.1 bc	15.4 b	3.2 cde
	Nebbiolo 1	368 e	541 d	6.68 cd	272 d	4.0 cd	nd	80.2 c	19.8 b	15.1 c	nd	55.6 c	8.2 bc	11.1 d	9.5 c	11.5 c	4.0 ab
	Nebbiolo 2	242 h	303 f	3.10 f	158 f	5.1 b	nd	83.7 b	16.3 c	14.9 c	nd	62.2 a	7.1 d	11.2 d	6.6 d	9.2 f	3.7 abc
	F	17,351	747	134	379	97		196	196	927		141	70	809	133	657	24.7
	Sign	***	***	***	***	***		***	***	***		***	***	***	***	***	***

All data are expressed as mg/g d.w. of freeze-dried extract except where indicated. The DPPH_AAE_ parameter is expressed as mg AAE/g extract d.w. EGC = (*−*)-epigallocatechin; EC = (*−*)-epicatechin; C = (+)-catechin; ECG = (*−*)-epicatechin-3-O-gallate ^a^ Different letters along the column discriminate the samples significantly different from one another (*p* < 0.05, Tukey’s test). ^b^ Significance: **, *** represent significance at *p* ≤ 0.01, 0.001. nd—not detected.

**Table 4:** Content in monomer, dimer, and trimer flavan-3-ols of the skin and seed extracts (concentrations of the single molecules expressed as mg/g d.w. of freeze-dried extract, concentration of each family of molecules expressed as μmol/g d.w. of freeze-dried extract). ANOVA results.

		C	EC	ECG	Σ Flavan-3-ols (μmol/g)	Dimer B1	Dimer B2	Dimer B3	Σ Dimers (μmol/g)	Trimer C1	Trimer C1 (μmol/g)
**Skins**	Muscat blanc	1.31 cd ^a^	0.67 e	0.15 c	7.13 e	0.94 cd	0.05 g	0.43 b	2.47 d	0.41 d	0.47 d
	Arneis	1.21 cd	1.08 d	0.10 d	6.13 de	0.56 g	0.25 ef	0.00 c	1.39 g	0.41 d	0.48 d
	Cortese	0.70 e	0.59 e	0.23 b	4.98 f	0.67 f	0.23 f	0.33 b	2.12 e	1.19 a	1.37 a
	Barbera	0.80 e	0.58 e	0.00 e	4.75 f	0.80 e	0.27 ef	0.00 c	1.86 f	0.17 e	0.20 e
	Grignolino	1.37 c	1.66 c	0.25 b	10.99 c	1.08 a	0.54 c	0.00 c	2.80 c	0.34 d	0.39 d
	Pinot noir 1	4.47 a	2.71 a	0.00 e	24.76 a	0.91 d	1.32 b	0.70 a	5.07 a	0.43 d	0.50 d
	Pinot noir 2	3.13 b	2.07 b	0.38 a	18.78 b	0.00 h	2.91 a	0.00 c	5.03 a	1.00 b	1.16 b
	Nebbiolo 1	0.63 e	1.06 d	0.00 e	5.86 f	1.04 ab	0.33 de	0.00 c	2.36 de	0.76 c	0.88 c
	Nebbiolo 2	1.12 d	1.43 c	0.15 c	9.15 d	1.00 bc	0.39 d	0.66 a	3.54 b	0.96 b	1.11 b
	F	1508	280	1155	1064	685	3655	279	789	156	156
	Sign	*** ^b^	***	***	***	***	***	***	***	***	***
**Seeds**	Muscat blanc	15.93 c	10.62 d	0.63 bc	92.91 d	2.54 c	2.03 cd	4.67 bc	15.97 d	2.89 c	3.34 c
	Arneis	14.05 d	13.79 c	0.62 bc	97.31 c	2.18 d	3.36 ab	2.49 de	13.88 e	0.66 g	0.77 g
	Cortese	6.23 f	5.53 f	0.49 cd	41.63 g	1.47 f	1.64 d	3.13 d	10.78 f	3.46 b	3.99 b
	Barbera	8.80 e	8.67 e	0.55 c	61.42 f	2.20 d	3.04 b	2.23 e	12.91 e	1.31 f	1.51 f
	Grignolino	3.46 h	4.90 f	0.44 cd	29.79 h	1.82 e	1.61 d	1.37 f	8.28 g	0.62 g	0.72 g
	Pinot noir 1	56.63 a	34.55 a	2.12 a	318.92 a	4.64 b	3.29 ab	6.60 a	25.12 a	2.58 d	2.98 d
	Pinot noir 2	24.16 b	18.75 b	0.76 b	149.56 b	5.16 a	3.55 a	4.40 c	22.66 b	2.23 e	2.57 e
	Nebbiolo 1	15.57 c	9.12 e	0.44 cd	86.07 e	2.53 c	2.48 c	5.21 b	17.66 c	5.14 a	5.93 a
	Nebbiolo 2	4.00 g	2.16 g	0.33 d	21.98 i	1.08 g	0.82 e	0.79 f	4.64 h	1.17 f	1.35 f
	F	39,560	3089	245	42,994	2089	112	241	667	839	839
	Sign	***	***	***	***	***	***	***	***	***	***

^a^ Different letters along the column discriminate the samples significantly different from one another (*p* < 0.05, Tukey’s test). ^b^ Significance: *** represents significance at *p* ≤ 0.001.

**Table 5:** Flavonols and phenolic acids in the skin and seed extracts (concentrations of the single molecules expressed as mg/g d.w., and concentrations of each family of molecules expressed as μmol/g d.w. of freeze-dried extract). ANOVA Results.

	Skins														Seeds			
	Gallic Acid (mg/g)	Gallic Acid (μmol/g)	Acid t-CafT	Acid c-CumT	Acid t-CumT	Acid t-FerT	Σ HCTA (μmol/g)	Quercetin	Q Glucor	Q Glucos	Kaempferol	K Glucor	K Glucos	Σ Flavonols (μmol/g)	Gallic Acid (mg/g)	Gallic Acid (μmol/g)	Acid t-CafT	Σ HCTA (μmol/g)
Muscat blanc	0.71 c ^a^	4.19 c	0.34 d	0.20 a	0.21 c	0.11 e	2.76 d	0.03 i	3.72 a	2.18 b	0.00 f	1.37 a	2.08 a	20.20 b	3.12 a	18.36 a	0.00 g	0.00 g
Arneis	0.61 d	3.61 d	0.15 g	0.19 b	0.00 f	0.09 f	0.77 h	0.21 f	2.92 c	4.06 a	0.08 e	0.76 b	1.77 b	21.38 a	1.30 e	7.66 e	0.00 g	0.00 g
Cortese	0.83 b	4.90 b	0.40 c	0.19 b	0.17 e	0.00 g	1.88 f	0.17 g	2.15 d	1.22 c	0.06 e	0.37 e	0.36 d	9.45 f	1.36 d	8.01 d	0.08 f	0.26 f
Barbera	0.81 b	4.79 b	0.70 a	0.17 c	0.32 a	0.38 a	5.30 a	2.68 a	3.19 b	0.82 e	0.41 a	0.42 d	0.16 e	19.52 c	1.56 b	9.16 b	0.45 a	1.45 a
Grignolino	0.69 cd	4.06 cd	0.11 h	0.16 d	0.19 d	0.15 d	2.12 e	1.22 e	0.56 h	0.48 f	0.26 c	0.13 h	0.07 ef	7.36 h	0.61 h	3.59 h	0.21 d	0.68 d
Pinot noir 1	0.26 f	1.56 f	0.00 i	0.00 e	0.00 f	0.00 g	0.00 i	0.10 h	1.64 e	0.98 d	0.07 e	0.00 i	0.98 c	8.31 g	1.43 c	8.43 c	0.09 e	0.29 e
Pinot noir 2	1.14 a	6.68 a	0.69 b	0.00 e	0.26 b	0.30 b	4.74 b	2.44 b	0.97 g	0.00 h	0.18 d	0.63 c	0.39 d	12.51 e	1.53 b	9.00 b	0.24 b	0.78 b
Nebbiolo 1	0.47 e	2.78 e	0.20 f	0.00 e	0.00 f	0.18 c	1.21 g	1.43 d	1.53 f	2.20 b	0.43 a	0.22 g	0.33 d	15.19 d	1.10 f	6.48 f	0.00 g	0.00 g
Nebbiolo 2	1.17 a	6.86 a	0.32 e	0.00 e	0.21 c	0.16 d	2.93 c	1.94 c	0.29 i	0.14 g	0.34 b	0.28 f	0.02 f	8.81 g	0.95 g	5.57 g	0.23 c	0.75 c
F	415	415	39,506	18,970	5438	1512	16,118	33,051	13,649	4002	967	2013	1087	3950	13,547	13,547	52,419	52,419
Sign	*** ^b^	***	***	***	***	***	***	***	***	***	***	***	***	***	***	***	***	***

^a^ Different letters along the column discriminate the samples significantly different from one another (*p* < 0.05, Tukey’s test). ^b^ Significance: *** represents significance at *p* ≤ 0.001.

**Table 6:** Correlation matrices between the DPPH parameter, the main polyphenolic compounds, and groups of compounds determined in the skin and seed extracts.

Seeds	Gallic Acid	Flavan-3-ols Monomers	Flavan-3-ols Dimers	C1 Trimer	HCTA	Condensed Tannins	Total Flavonoids	GAE Index	DPPH_AAE_	
Gallic acid	**1**									
Flavan-3-ols monomers	0.182	**1**								
Flavan-3-ols dimers	0.334	**0.869**	**1**							
C1 trimer	0.241	0.181	0.414	**1**						
HCTA	−0.26	−0.212	−0.238	**−0.481**	**1**					
Condensed tannins	0.342	**0.700**	**0.764**	0.064	**−0.517**	**1**				
Total flavonoids	**0.495**	**0.496**	**0.665**	0.194	**−0.520**	**0.917**	**1**			
GAE index	**0.622**	**0.557**	**0.688**	0.233	**−0.560**	**0.900**	**0.970**	**1**		
DPPH_AAE_	**0.675**	0.195	0.402	0.269	**−0.601**	**0.708**	**0.895**	**0.880**	**1**	
**Skins**	Gallic acid	Flavan-3-ols monomers	Flavan-3-ols dimers	C1 trimer	Flavonols	HCTA	Condensed tannins	Total flavonoids	GAE index	DPPH_AAE_
Gallic acid	**1**									
Flavan-3-ols monomers	**−0.502**	**1**								
Flavan-3-ols dimers	0.091	**0.701**	**1**							
C1 trimer	**0.501**	−0.009	0.261	**1**						
Flavonols	−0.053	−0.231	**−0.504**	−0.334	**1**					
HCTA	**0.737**	−0.338	0.025	0.022	0.222	**1**				
Condensed tannins	**0.531**	−0.181	0.356	**0.521**	−0.368	0.247	**1**			
Total flavonoids	**0.525**	0.088	**0.562**	0.356	−0.291	**0.494**	**0.848**	**1**		
GAE index	**0.493**	−0.029	0.444	**0.497**	−0.212	0.136	**0.895**	**0.770**	**1**	
DPPH_AAE_	**0.613**	−0.461	−0.029	0.272	0.122	0.425	**0.682**	**0.532**	**0.754**	**1**

XLSTAT 2019 was used to calculate the correlations. Values in bold are different from 0 with a significance level alpha = 0.05.

**Table 7:** Regression lines between the DPPH parameter (variable Y) and the main classes of polyphenolic compounds (variable X) determined in the skin and seed extracts.

Skins			Seeds		
x Variables	Regression Equation	*R* ^2^	x Variables	Regression Equation	*R* ^2^
GAE index	y = 0.007x + 1.225	0.569	GAE index	y = 0.021x + 1.469	0.775
gallic acid (GA)	y = 0.603x + 1.806	0.376	gallic acid (GA)	y = 1.326x + 3.879	0.456
flavan-3-ols monomers	y = −0.009x + 2.818	0.213	flavan-3-ols monomers	y = 0.017x + 6.639	0.038
flavan-3-ols dimers	y = −0.037 + 2.604	0.001	flavan-3-ols dimers	y = 0.496x + 5.031	0.162
C1 trimer	y = 1.111x + 2.337	0.074	C1 trimer	y = 1.275x + 6.188	0.073
flavonols (Fl)	y = 0.014 + 2.491	0.015			
condensed tannins (CT)	y = 0.010x + 1.613	0.466	condensed tannins (CT)	y = 0.019x + 1.366	0.502
HCTA	y = 0.407x + 2.287	0.181	HCTA	y = −9.934x + 8.483	0.361
Fl (x_1_) + CT (x_2_)	y = 0.048x_1_ + 0.030x_2_ + 1.106	0.627	GA (x_1_) + CT (x_2_)	y = 0.962x_1_ + 0.015x_2_ + 0.364	0.714

**Table 8:** Total polyphenols (GAE index) and condensed tannins in the skin extracts—Fraction 2B and 3 (data expressed as mg/g d.w. of freeze-dried extract).

					Total Monomeric Composition				Extension Units				Terminal Units		
		GAE	Condensed Tannins	mDP	% EGC	% EC	% C	% ECG	% EGC-p	% EC-p	% C-p	% ECG-p	% EC	% C	% ECG
**Skins: fraction 2B**	Muscat blanc	20.2 d ^a^	3.8 fg	2.6 d	3.6 a	65.7 d	34.3 d	9.8 e	3.6 a	43.8 d	9.5 cd	4.4 f	8.5 g	24.7 b	5.4 c
	Arneis	18.2 e	3.9 f	2.6 d	0 b	70.9 c	29.1 e	8.7 f	0 b	48.4 b	8.5 cde	4.5 f	13.8 c	20.6 c	4.3 d
	Cortese	27.1 b	7.1 d	2.9 b	0 b	73.0 b	27.0 f	18.2 a	0 b	46.3 c	9.8 c	9.3 a	8.5 g	17.2 e	8.9 a
	Barbera	12.7 g	3.7 fg	2.6 d	0 b	73.8 b	26.2 f	11.9 c	0 b	46.8 c	7.6 e	6.6 c	15.1 a	18.6 d	5.3 c
	Grignolino	27.9 b	10.2 b	3.0 a	0 b	77.4 a	22.6 g	12.0 c	0 b	53.4 a	8.2 de	5.6 d	12.1 d	14.4 f	6.4 b
	Pinot noir 1	18.3 e	4.7 e	2.2 f	0 b	51.9 g	48.1 a	8.2 g	0 b	29.3 f	22.3 ab	2.7 g	14.4 b	25.9 a	5.5 c
	Pinot noir 2	34.7 a	15.8 a	2.9 c	0 b	58.2 e	41.8 c	10.4 d	0 b	36.4 e	22.9 a	5.1 e	11.4 e	18.9 d	5.3 c
	Nebbiolo 1	16.4 f	3.7 g	2.4 e	0 b	53.5 f	46.5 b	13.5 b	0 b	29.8 f	21.3 b	6.9 b	10.2 f	25.2 ab	6.6 b
	Nebbiolo 2	25.7 c	8.2 c	2.6 d	0 b	73.4 b	26.6 f	10.4 d	0 b	48.4 b	8.6 cde	5.1 e	14.6 ab	18.0 de	5.3 c
	F	1198	14152	346	17729	1415	1415	1571	17729	1350	673	2025	750	485	595
	Sign	*** ^b^	***	***	***	***	***	***	***	***	***	***	***	***	***
**Skins: fraction 3**	Muscat blanc	108.2 f	20.0 i	7.4 fg	0.93 e	64.3 e	35.7 a	9.1 h	0.9 e	53.0 cd	25.4 a	7.1 h	1.3 f	10.3 a	1.9 c
	Arneis	129.3 d	42.3 g	8.5 c	3.56 a	88.1 a	11.9 e	11.91 f	3.6 a	70.3 a	4.6 e	9.9 f	2.3 d	7.4 c	2.0 c
	Cortese	138.1 c	54.1 d	9.5 b	0 h	71.5 c	28.5 c	20.4 a	0 h	49.6 e	22.3 b	17.6 a	1.5 ef	6.2 d	2.8 a
	Barbera	69.4 g	29.9 h	7.6 ef	0.73 f	67.8 d	32.2 b	15.4 b	0.7 f	48.3 e	24.4 a	13.5 b	3.4 b	7.8 b	1.9 c
	Grignolino	164.1 b	73.7 b	8.8 c	0 h	87.5 a	12.5 e	14.9 c	0 h	69.3 a	6.7 d	12.7 c	3.3 b	5.9 d	2.2 b
	Pinot noir 1	135.3 c	42.8 f	8.1 d	1.18 d	68.1 d	31.9 b	10.5 g	1.2 d	53.7 c	24.1 a	8.6 g	2.6 c	7.8 b	1.9 c
	Pinot noir 2	202.2 a	89.5 a	7.8 e	0.52 g	73.13 b	26.9 d	13.5 d	0.5 g	55.7 b	19.5 c	11.5 d	3.4 b	7.4 c	2.0 c
	Nebbiolo 1	121.3 e	50.9 e	10.4 a	3.34 b	72.01 bc	28.0 cd	13.3 de	3.3 b	53.9 c	21.8 b	11.4 d	1.5 e	6.2 d	1.8 c
	Nebbiolo 2	124.4 e	56.4 c	7.3 g	2.40 c	71.20 c	28.8 c	13.2 e	2.4 c	51.5 d	21.5 b	10.9 e	4.1 a	7.3 c	2.4 b
	F	2784	85,736	385	2957	1000	1000	6067	2957	711	889	13,738	842	438	77
	Sign	***	***	***	***	***	***	***	***	***	***	***	***	***	***

^a^ Different letters along the column discriminate the samples significantly different from one another (*p* < 0.05, Tukey’s test). ^b^ Significance: *** represents significance at *p* ≤ 0.001.

**Table 9:** Total polyphenols (GAE index) and condensed tannins in the seed extracts—Fraction 2B and 3 (data expressed as mg/g d.w. of freeze-dried extract).

					Total Monomeric Composition			Extension Units			Terminal Units		
		GAE	Condensed Tannins	mDP	% EC	% C	% ECG	% EC-p	% C-p	% ECG-p	% EC	% C	% ECG
**seeds: fraction 2B**	Muscat blanc	106.3 d ^a^	68.1 c	3.1 cd	67.0 cd	33.0 ab	15.4 b	42.2 c	18.2 ab	7.7 b	9.4 fg	14.7 bc	7.7 b
	Arneis	90.1 e	51.3 d	3.04 f	80.1 a	19.9 d	6.7 g	55.0 a	8.6 d	3.5 h	18.3 a	11.3 f	3.3 g
	Cortese	79.2 f	51.1 d	3.5 a	72.4 bc	27.6 bc	16.5 a	46.9 abc	16.3 abcd	8.0 a	9.0 g	11.3 f	8.5 a
	Barbera	76.4 g	40.4 f	3.1 de	77.0 ab	23.0 cd	8.9 f	51.4 ab	10.8 bcd	5.6 f	16.7 b	12.2 e	3.3 g
	Grignolino	56.1 h	31.2 g	3.3 b	66.8 cd	33.2 ab	11.8 d	41.9 c	21.1 a	6.2 c	13.0 d	12.1 e	5.6 e
	Pinot noir 1	114.2 b	82.1 b	3.2 c	63.6 d	36.4 a	12.3 c	41.0 c	21.3 a	6.0 d	10.2 ef	15.1 b	6.4 d
	Pinot noir 2	121.3 a	90.4 a	3.1 ef	73.6 abc	26.4 bcd	11.0 e	51.6 ab	10.0 cd	5.9 e	11.0 e	16.3 a	5.2 f
	Nebbiolo 1	108.4 c	49.3 e	3.0 g	68.3 cd	31.7 ab	12.4 c	43.6 bc	17.4 abc	5.3 g	12.4 d	14.3 cd	7.1 c
	Nebbiolo 2	35.2 i	17.2 h	2.9 g	74.0 abc	26.0 bcd	11.9 d	48.0 abc	12.1 bcd	5.6 f	14.1 c	13.9 d	6.3 d
	F	19291	38722	272	15.3	15.3	32842	10.5	11	7363	337	318	4120
	Sign	*** ^b^	***	***	***	***	***	***	***	***	***	***	***
**seeds: fraction 3**	Muscat blanc	306.3 c	156.2 c	8.1 b	82.7 e	17.3 b	16.7 b	63.1 f	10.6 b	14.0 b	3.0 g	6.7 b	2.7 ab
	Arneis	329.2 b	108.1 f	7.9 bc	89.4 a	10.6 f	7.2 g	76.2 a	6.0 e	5.1 g	6.0 b	4.6 f	2.1 cd
	Cortese	334.2 ab	218.3 a	10.4 a	86.5 c	13.5 d	20.2 a	64.2 e	9.0 c	17.2 a	2.1 h	4.5 f	3.1 ab
	Barbera	188.4 e	82.4 g	7.0 d	87.0 bc	13.0 de	10.3 f	69.7 c	7.6 d	8.4 f	6.9 a	5.4 e	1.9 d
	Grignolino	239.2 d	125.3 d	8.1 b	87.7 b	12.3 e	12.2 e	71.0 b	7.0 d	9.7 d	4.4 d	5.3 e	2.6 bc
	Pinot noir 1	305.3 c	178.3 b	8.1 b	87.9 b	12.1 e	14.7 c	69.8 c	5.9 e	12.0 c	3.4 f	6.2 c	2.7 ab
	Pinot noir 2	344.1 a	179.1 b	6.7 e	84.4 d	15.6 c	10.8 f	68.6 d	7.7 d	8.7 e	5.0 c	7.9 a	2.1 cd
	Nebbiolo 1	241.1 d	122.2 e	7.7 c	84.8 d	15.2 c	12.9 d	68.1 d	9.3 c	9.7 d	3.8 e	6.0 d	3.2 a
	Nebbiolo 2	131.4 f	55.3 h	7.3 d	69.5 f	30.5 a	12.5 de	52.1 g	24.4 a	9.6 d	4.8 c	6.0 cd	2.9 ab
	F	1617	46,532	384	1061	1061	1669	1811	1095	7408	560	893	23.2
	Sign	***	***	***	***	***	***	***	***	***	***	***	***

^a^ Different letters along the column discriminate the samples significantly different from one another (*p* < 0.05, Tukey’s test). ^b^ Significance: *** represents significance at *p* ≤ 0.001.

**Table 10:** Monomer flavan-3-ols in the skin and seed extracts—Fraction 2A (data expressed as mg/g d.w. of freeze-dried extract).

		Muscat Blanc	Arneis	Cortese	Barbera	Grignolino	Pinot Noir 1	Pinot Noir 2	Nebbiolo 1	Nebbiolo 2	F	Sign
skins	C	0.45 c ^a^	0.24 f	0.00 i	0.12 g	0.40 e	1.75 a	0.85 b	0.09 h	0.42 d	123245	*** ^b^
	EC	0.14 d	0.15 d	0.06 e	0.03 ef	0.30 c	0.81 a	0.43 b	0.00 f	0.11 d	886	***
seeds	C	10.64 c	8.76 d	4.20 e	4.18 e	1.37 f	43.52 a	15.37 b	10.86 c	0.94 g	103282	***
	EC	7.16 d	7.42 c	3.64 f	3.37 g	1.35 h	24.33 a	7.71 b	5.42 e	0.32 i	322100	***
	ECG	0.00 g	0.04 e	0.16 b	0.03 f	0.03 f	0.38 a	0.10 c	0.05 d	0.00 g	30151	***

^a^ Different letters along the row discriminate the samples significantly different from one another (*p* < 0.05, Tukey’s test). ^b^ Significance: *** represents significance at *p* ≤ 0.001.

## Data Availability

The data used to support the findings of this study can be made available by the corresponding author upon request.
